# Evolving convolutional neural network parameters through the genetic algorithm for the breast cancer classification problem

**DOI:** 10.1177/0037549721996031

**Published:** 2021-03-05

**Authors:** Khatereh Davoudi, Parimala Thulasiraman

**Affiliations:** Department of Computer Science, University of Manitoba, Canada

**Keywords:** Evolutionary machine learning, breast cancer, computer-aided diagnosis systems, deep learning, convolutional neural network, back-propagation, genetic algorithm

## Abstract

Breast cancer is the most frequently diagnosed cancer and the leading cause of cancer mortality in women around the world. However, it can be controlled effectively by early diagnosis, followed by effective treatment. Clinical specialists take the advantages of computer-aided diagnosis (CAD) systems to make their diagnosis as accurate as possible. Deep learning techniques, such as the convolutional neural network (CNN), due to their classification capabilities on learned feature methods and ability of working with complex images, have been widely adopted in CAD systems. The parameters of the network, including the weights of the convolution filters and the weights of the fully connected layers, play a crucial role in the classification accuracy of any CNN model. The back-propagation technique is the most frequently used approach for training the CNN. However, this technique has some disadvantages, such as getting stuck in local minima. In this study, we propose to optimize the weights of the CNN using the genetic algorithm (GA). The work consists of designing a CNN model to facilitate the classification process, training the model using three different optimizers (mini-batch gradient descent, Adam, and GA), and evaluating the model through various experiments on the BreakHis dataset. We show that the CNN model trained through the GA performs as well as the Adam optimizer with a classification accuracy of 85%.

## 1. Introduction

According to the global cancer statistics 2018,^1^ breast cancer, with 24.2% of total cancer cases, is the most regularly diagnosed cancer type and the main cause of cancer mortality among women. However, it is one of the few cancers that can be controlled effectively by early-stage diagnosis. Despite the significant development of non-invasive breast imaging modalities, such as mammography and ultrasound, invasive medical screening is the gold standard for final breast cancer diagnosis in clinical scenarios. Invasive techniques refer to the histological assessment of breast biopsy images by a pathologist to classify the breast images into benign or malignant cases based on specific features, such as nuclei characteristics, density, variability, and spatial arrangement.

However, due to the inherent complexity of breast biopsies, analysis of these images is a complicated and highly time-consuming task, affected by different factors such as level of knowledge, experience, attention, and fatigue of specialists.^2–5^ As a result, to overcome the shortcomings of human interpretation, computer-aided diagnosis (CAD) systems have become essential and crucial in the breast cancer classification problem to facilitate the diagnosis process and increase survival chances. Classification is the most critical component of CAD systems. Different machine learning techniques have been broadly adopted in the classification step of CAD systems.^6–8^ The classification accuracy of machine learning techniques is highly dependent on the quality of the extracted features. The feature extraction methods can be divided into two separate categories: *hand-crafted* and *learned*. Hand-crafted feature selection approaches, such as support vector machines (SVMs)^9^ and artificial neural networks (ANNs),^10^ extract the features using manually designed algorithms based on expert knowledge of the problem, which is a time-consuming and low-efficiency process.^5,11^ Histopathological breast images are complex and fine-grained images with high diversity and inhomogeneity of color distribution. Therefore, the convolutional neural network (CNN), a subset of learned machine learning techniques, is becoming a valuable alternative. The CNN is a particular type of deep, feed-forward neural network that learns the features directly from the image dataset through a training procedure. Despite the recent development of CAD systems, breast cancer diagnosis remains a crucial health issue, and research is still on-going to improve the accuracy of CAD systems.

**Figure 1.**
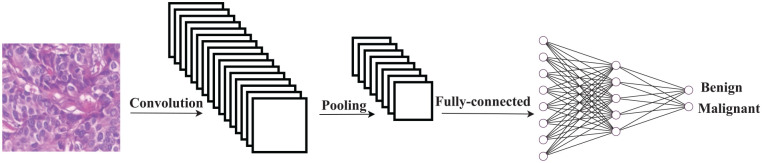
An overview of the convolution neural network for the breast cancer classification task.

This study focuses on the CNN for the classification of breast cancer histology images. The CNN is inspired by the biological neural network of the human brain. The technique is based on the idea that the system can learn from previous data. The CNN includes several hidden layers, such as convolution, pooling, and fully connected layers between the input and output layers, as shown in Figure 1. The precision of the extracted features in the CNN is highly dependent on the weights of the network, including the weights of all convolution filters, as well as the weights of connecting edges in the fully connected layer. Consequently, these weights play a crucial role in classification accuracy. During the training phase of the model, the weights are updated continuously to achieve a minimum classification error rate.

Back-propagation is the most frequently used technique for updating the weights in neural networks.^2,12^ This algorithm uses an optimization technique called gradient descent to update the weights. The gradient of the model parameters is re-sampled, repetitively, in the backward direction of the network weights, to find a set of weights that minimizes the classification error value.^13^ The disadvantage of the back-propagation algorithm, however, is that it requires high convergence time and may get stuck in local optima. Trapping in local optima means that during the process of finding the weights, which minimize the classification error value, the back-propagation algorithm may find a weight with smaller error value at nearby points, which is not necessarily the smallest one at all other feasible points.^13–15^ To overcome the shortcomings of the back-propagation strategy, the genetic algorithm (GA),^16^ a well-known global optimization technique inspired by the process of natural selection, has been proposed for optimizing the weights of the neural network.^17–19^

This is a feasibility study of hybridizing and evolutionary algorithm with machine learning models for the breast cancer classification problem. To the best of our knowledge, there is no scientific work using the GA to optimize the parameters in the CNN for histopathological breast image classification; therefore, in this work, we apply the GA instead of back-propagation to classify breast biopsy images. We compare the performance of the GA-based CNN with mini-batch gradient descent and Adam optimizers. The models are compared with metrics such as classification accuracy, recall, precision, *F*_1_-score, and execution time. We use the BreakHis dataset containing breast cancer histopathological images for training and testing our proposed technique.^20^

This work makes the following contribution:

- Optimize the CNN weights for histopathological breast image classification problem using the GA search heuristic;
- Design and develop a GA-CNN model for binary classification (benign (non-cancerous) and malignant (cancerous)) of histopathological breast images;
- Train the model using different optimizers, namely mini-batch gradient descent, Adam, and the GA;
- Evaluate the model through various experiments on the BreakHis dataset.^3^

The rest of this paper is organized as follows. Section 3 provides the background. Section 4 describes our proposed model. Datasets and evaluation methods are addressed in Section 5. Experimental results are discussed in Section 6. Section 7 presents the discussion and conclusion. Future work is presented in Section 8.

## 2. Literature review

In this section, the literature review on breast cancer classification with respect to machine learning and evolutionary techniques is discussed.

### 2.1. Machine learning

Machine learning is one of the most investigated research areas for breast cancer classification problem. Singh et al.^21^ proposed a method for breast cancer mass identification and calcification in breast mammograms. They used a combination of *K-means* and *fuzzy C-means* clustering to diagnose breast cancer. The experimental results illustrate that this proposed approach can facilitate the early breast cancer diagnosis process. Lashkari and Firouzmand^22^ compared a supervised technique called the Adaboost method to the unsupervised fuzzy C-means algorithm for breast thermogram image classification. They concluded that the fuzzy C-means method, with 75% classification accuracy, can be a suitable solution for unsupervised classification.

The capability of the SVM for supervised binary classifications has allowed the technique to be broadly used for breast cancer diagnosis. Akay^9^ used the SVM to provide a breast cancer diagnosis system. The author proposed an improved classification system that combined the SVM with feature selection to classify breast images. Their proposed model showed a classification accuracy of 98.53% on the WBCD dataset.^23^ Shirazi and Rashedi^24^ proposed a model based on the SVM and a mixed gravitational search algorithm for tumor detection in breast mammography images. The main objective of this work was to improve the SVM classification accuracy by reducing the number of features. The experimental results of this work showed that the SVM with the mixed gravitational search method obtained 93.10% accuracy.

The ANN allows computers to learn and make decisions based on previous experiences in a human-like manner. This machine learning technique is widely and efficiently used for the breast cancer classification problem. Earlier work on the ANN with back-propagation training approach was widely used for mammography.^10^ Nahato et al.^25^ proposed the RS-BPNN, which combined the rough set indiscernibility relation method with the gradient descent back-propagation neural network (BPNN) for breast tumor classification. The indiscernibility relation method was used to handle missing values to obtain a reliable dataset and select attributes from clinical data, while the BPNN was used as a classifier on the dataset. The RS-BPNN provided 98.6% classification accuracy. In another study, Bhattacherjee et al.^26^ trained a BPNN to obtain an accuracy of 99.27%. A performance comparison of the SVM and the ANN was done by Ali and Feng^27^ for the binary classification of the WDBC dataset. The results of this study showed that the ANN approach outperformed that of the SVM in terms of accuracy, precision, and efficiency for the classification of breast images as either benign or malignant cases. In a recent study, Saritas and Yasar^28^ compared the performance of the ANN and naive Bayes classifiers and reported an accuracy of 86.95% and 83.54% with the ANN and naive Bayes algorithms, respectively.

Gupta and Raza^29^ proposed a new methodology that can optimize the number of hidden layers and their respective neurons for a deep feed-forward neural network using a combination of combination of Tabu search and gradient descent with a momentum back-propagation training algorithm. The experimental results of this study show better generalization ability of the optimized networks.

Although most of the discussed conventional classification methodologies provide an acceptable classification accuracy, their performance depends on proper data representation and hand-crafted feature extraction. This is a complex, challenging, and time-consuming task. The other alternatives that have gained momentum are learned feature techniques, such as *deep learning*. The CNN, as a well-known class of deep neural networks used for the visual imagery analysis, is widely employed by different researchers to classify complex breast images. Fakoor et al.^30^ used a deep learning model to increase the performance of cancer detection and diagnosis by forming the features automatically, based on gene expression data. The experimental results showed that deep learning produced better performance than previous classification methods. Wang et al.^12^ studied metastatic breast cancer diagnosis using a combination of deep learning and human pathologists’ diagnoses. The authors showed the power of deep learning to increase the accuracy of diagnosis. Spanhol et al.^3^ used the CNN to classify breast histopathological images, achieved by microscopic examination of breast biopsies. Their proposed methodology was based on image patch extraction for training the CNN system, followed by combining the image patches to classify the histopathological images. The results of this study on the BreakHis breast cancer dataset^20^ indicated that the CNN performed better compared to hand-crafted classifiers.

Bayramoglu et al.^31^ proposed a CNN-based approach to automate the diagnosis of breast cancer in histopathology images, independent of their magnifications. A comparison of the classification performance of this method and previous models, in which hand-crafted feature extraction techniques were used, showed that the performance improved with the CNN model. In another study, a CNN model for multi-classification of the breast biopsy images was proposed by Araújo et al.^2^ to cover the shortcomings of conventional feature extraction classification techniques. This model achieved a classification accuracy of 77.8% for the multi-classification of breast biopsies. Nawaz et al.^32^ created a CNN-based multi-class breast cancer classification. Using the DenseNet and BreakHis training datasets, their proposed approach provided a high classification accuracy of 95.4%. An ensemble deep learning-based method was employed by Kasani et al.^33^ for binary classification of histopathological biopsy images into malignant and benign cases. Experimenting on multiple datasets, including BreakHis, ICIAR, PatchCamelyon, and Bioimaging, the ensemble deep learning-based method achieved 83.10–98.13% classification accuracy.

### 2.2. Evolutionary genetic algorithm

Theoretically, it is expected that the CNN should outperform other machine learning techniques for breast biopsy image classification. An important reason for any probable failure to obtain high classification accuracy could be due to the training algorithm used for updating the network weights during the learning process. The back-propagation technique is the most regularly used approach for updating the weights of the network during the training process of the neural network. However, because of the disadvantages of the back-propagation method, such as the inability to escape local optima and high convergence time, as well as sensitivity to noisy data, research on alternative training methods is pursued to overcome these challenges. Evolutionary techniques, such as the GA, are some of the most investigated alternative techniques to overcome the weaknesses of the back-propagation technique in the neural network.^34,35^

The GA is a bio-inspired optimization technique that follows the process of natural selection of genes in nature. This technique is well-suited for generating accurate solutions for global optimization and search problems through its operations, such as evaluation, selection, crossover, and mutation.^16^ In the literature, the GA has been used either for updating the weights of the neural network or learning the network structures.^36^ In one of the earlier works, Montana and Davis^14^ used the GA to train a feed-forward neural network. The authors showed that in comparison to the BPNN, the GA improved the accuracy by optimizing the weights during the neural network learning process. Ahmad et al.^37^ compared the performance of gradient descent and the GA-based ANN applied to cancer and diabetes benchmark datasets. They also tested the effect of the crossover operation on the GA performance. Their results illustrated better classification GA accuracy on the cancer dataset versus a higher accuracy of gradient descent on diabetes images.

Belciug and Gorunescu^15^ proposed a combination of a neural network and GA to classify a patient dataset into malignant or benign and recurrent or non-recurrent breast cancer cases. They designed a multi-layer perceptron using the GA to update the network weights during the training phase. The results of this study indicated that the classification accuracy of the hybrid approach outperformed the traditional BPNN. Bhardwaj and Tiwari^35^ used genetic programming (GP) to optimize a neural network for breast cancer diagnosis. GP was used to optimize the weights and network architecture. The crossover and mutation functions were modified to expand the search area. The results showed 99.26% classification accuracy using 10-fold cross-validation.

The GA is also widely used to improve the performance of deep learning approach. Young et al.^38^ proposed to use the GA for automating model selection in deep learning. The authors concluded that this approach could be more powerful than a random search for finding the best network topology. Ijjina and Chalavadi^39^ proposed a model for human action recognition. They minimized the classification error rate by initializing the network weights using the GA. They used a gradient descent algorithm for CNN classifiers during fitness evaluations of GA chromosomes. The experimental results of this study demonstrated that the combination of gradient descent and the GA provided a recognition accuracy of 99.9%. In another work, Such et al.^34^ used a gradient-free GA to optimize the weights of a deep neural network. The proposed method easily evolved networks with more than 4 million parameters and trained the system faster.

Martin et al.^40^ proposed an evolutionary approach for automatic deep neural network parametrization, achieving a classification accuracy of 98.93%. Sun et al.^18^ proposed using the GA to optimize both architectures and initial weight values of a deep CNN for image classification problems. The results of this study indicated a significant superiority over state-of-the-art algorithms in terms of classification accuracy and the number of weights. Optimizing the weights of the ANN using the GA for the image classification problem was discussed by Gad and Gad.^17^

To the best of our knowledge, there is no such study combining the GA and the CNN to improve histopathological breast cancer classification accuracy. Thus, in this work, the proposed approach by Gad and Gad^17^ is extended using a combination of the CNN and the GA to classify breast biopsy images.

## 3. Background

In this section, we discuss the background needed for our proposed approach. In particular, we discuss the basics of the CNN and the GA, two techniques used in this study. This is followed by a detailed discussion of our proposed approach, evolving the CNN through the GA in the next section for breast image classification.

### 3.1. Convolutional neural network

The CNN is a class of deep learning that is inspired by the operation of biological neurons in the human brain. This method is highly suitable for working with two-dimensional image classification tasks. The CNN accepts the images as input and extracts the required features automatically without any need for hand-crafted feature extraction methods. The CNN consists of the following layers: input, hidden, and output. Hidden layers contain convolution, pooling, flattening, and fully connected layers that transform the input data to the output layer accurately.^17^ The convolution layer includes multiple filters of the network weights. Also, the fully connected layer has a semantic group of nodes that are connected via weighted edges to the nodes in the following layers and previous layers. Finding the best set of weights for these convolution filters and connection edges plays a crucial role in the training process of the neural network. In the CNN, the input vector is transformed with a set of weights similar to a linear function, as shown in the following equation:

(1) y=w·x+b

In this equation, y , x , and w refer to the output, input, and weight, respectively. The bias term, b , is a constant added to a linear equation to increase the flexibility of the model.

An *activation function* calculates the outputs of the network based on inputs. This function will be applied to the above linear equation (1). The activation function decides whether a neuron should be activated (or not activated) based on the weighted sum and bias of the neuron. There are different types of activation functions, such as the linear regression model, non-linear models, Sigmoid functions, and Rectified Linear Unit (ReLU) activation functions. Two of the most commonly used activation functions are Sigmoid and ReLU activation functions. The Sigmoid (σ) function is represented by the mathematical equation given in Equation (2). It takes real values and projects them between 0 and 1. Note that the gradient of the function for the tail values is close to zero. The training process, would, therefore, fail for the features close to this region. The ReLU function is defined as *y* = max(0, *x*). In comparison to the Sigmoid function, the ReLU considerably improves the convergence rate of the training process due to its linear and non-saturating form. Also, the ReLU is not computationally intensive. However, the disadvantage is that since the ReLU eliminates all the negative information, it is not suited for all datasets and architectures. In this paper, we use the sigmoid function:

(2) $$y=\sigma (x)=\frac{1}{1+e^{-x}}$$

In what follows, each layer of the CNN in Figure 1 is briefly explained. The convolutional layer is the main component of the CNN. This layer converts the input image to a map of features by performing a linear operation called *convolution*. To extract the differences between various images, a group of filters are applied to these images. These filters convolve the input and pass the result to the next layer by applying a dot product *convolution* over the whole size of each image. The parameters of these filters (i.e., the weights) are initialized randomly and need to be learned by the network subsequently. After the convolutional layer, a pooling layer is added to the network. In this step, a pooling operation will be selected to be applied to feature maps aimed at reducing the number of features. The size of this filter is less than the size of the feature map; specifically, it is usually 2×2 pixels. *Average Pooling*, calculating the average of each patch of the feature matrix, and *Maximum Pooling*, computing the maximum value of each patch of the feature matrix, are two common types of pooling functions.

After the convolution and pooling steps, the entire obtained feature map matrix is transformed into a single vector using a procedure called *flattening*. This vector is then fed to the fully connected layer for further processing. After flattening, the obtained feature vector is sent to the fully connected layer as its input. This input vector, then, is connected to the output layer via a fully connected hidden layer. At the end of this process, the classification error rate is calculated using the predicted and actual classes. The recursive training process will continue until the highest classification accuracy is found.

Based on the definition of the CNN, filters and neurons carry a set of weights. The process of adjusting these weights using available datasets is named the training process. These weights are initialized randomly at the beginning and are updated using different optimization techniques in a recursive process. Back-propagation is one of the most commonly used techniques in the CNN training process. Two instances of back-propagation approaches are explained below.

*Gradient descent*: a vector indicating the direction with the largest changing value at one point refers to the gradient or the rate of change of a function. Gradient descent is frequently used in neural networks to define the training process of the networks. To quantify the capacity of the architecture in the approximation of the ground truth labels for all training inputs, we define a *loss function*. The number of correctly classified images could be used as a simple loss function. The gradient descent algorithm is used to minimize the loss function J(w) , as shown in Algorithm 1.^41^ The value of J(w) has to be made as small as possible to achieve the highest classification accuracy. Here, w is the network weight and α is the learning rate.

**Table:** 

**Algorithm 1.** Gradient descent.
Initializing the network weights randomly; Adjusting the weights to decrease the loss function: **for k=0,1,…,n do** $g_{k}\leftarrow \nabla J(w_{k})$ ; $w_{k+1}\leftarrow w_{k}-\alpha g_{k}$ ; **end for**

For large datasets, batch gradient descent is computationally intensive, since the whole dataset has to be trained at once. To overcome this problem, we can take the advantage of stochastic gradient descent, which selects a subset (or batch) of training data randomly to train the model. Stochastic gradient descent often converges much faster compared to gradient descent since it does not need to train the whole dataset at the same time. Mini-batch gradient descent, in which the batch size is set to greater than one and smaller than the whole dataset, is the most frequently used variant of gradient descent. A batch size of 32 is a good default batch value for mini-batch gradient descent.^42^ However, all gradient-based approaches may trap at local minima rather than finding global minima.

*Adam*: a constant learning rate is considered during the training process for all the weights and biases in both gradient descent and stochastic gradient descent techniques. Choosing the proper learning rate is fairly difficult because this rate changes the learning process significantly. Choosing a small constant learning rate that gives stable convergence is a viable solution, although this increases the computation time. To overcome this problem, the learning rate could be set as a small value for some initial epochs and then a smaller value is chosen as convergence slows down for the rest of the training process. This approach uses the first and second derivatives of the weights to define an adaptive learning rate for all the parameters changing over time.

The *Adaptive Moment Estimation* (Adam) algorithm is an extension of the stochastic gradient descent optimization approach, proposed by Kingma and Ba^43^ in 2014. This algorithm follows the idea of computing specific learning rates for each parameter. Adam uses the first-order gradient-based optimization. This is a straight-forward and efficient method with low memory usage. For cases with a large dataset, Adam fairly speeds up the training process. Although Adam can train models faster than other techniques, it may still get stuck in local optima.^44^

### 3.2 Genetic algorithm

The *GA* is a class of evolutionary algorithms that follows Darwin’s natural theory of evolution. It is a powerful technique for solving different search problems by finding globally optimal solutions.^45^

The first step in the GA is generating an *initial population* of individuals (or solutions) randomly. This helps in incorporating a diverse range of possible solutions for the problem. The variables that define the character of an individual are called *genes*. A collection of genes form a *chromosome*, that is, the solution to the search problem. Finally, a set of chromosomes form the initial population. There are different types of gene representations in the GA, such as binary, real-coded, or integer representation. The *evaluation* of an individual is done using an evaluation operator, the *fitness function*. The fitness function measures the ability of an individual to generate an optimal solution. In this phase, the fitness value of each individual is calculated and sorted. As a result, individuals with better fitness values will receive more opportunities to be selected for reproducing the offspring in the next generations. Fitness functions differ depending on the application and the search problem. For example, the classification error is a common fitness operation for different types of classification problems.

After calculating the fitness values, the *selection* phase is executed. In the selection phase, the mating process is determined—that is, which individuals should mate in producing the next generation. Individuals with better fitness scores are selected for passing their genes to the next generation. The *crossover* operation is considered as one of the most critical components in the GA. The action of this genetic operation is generating two new children chromosomes using the genes of two parent chromosomes. Among the different types of crossover operations, the single-point crossover is the most frequently used approach. In a single-point crossover, a random crossover point is selected in each of the individuals. Then, the genes of each side are swapped between parents to generate new offspring.^46^

After producing the new offspring, some of the genes in the new individuals are *mutated* to increase the diversity in the population from one generation to another. Increasing the diversity of the population can produce stronger individuals or solutions.^47^ Flip bit, inversion, and random mutation are some of the most common types of mutation operations used for real-coded or binary representation in the GA.^48^

## 4. Evolving the convolutional neural network through the genetic algorithm

**Figure 2.**
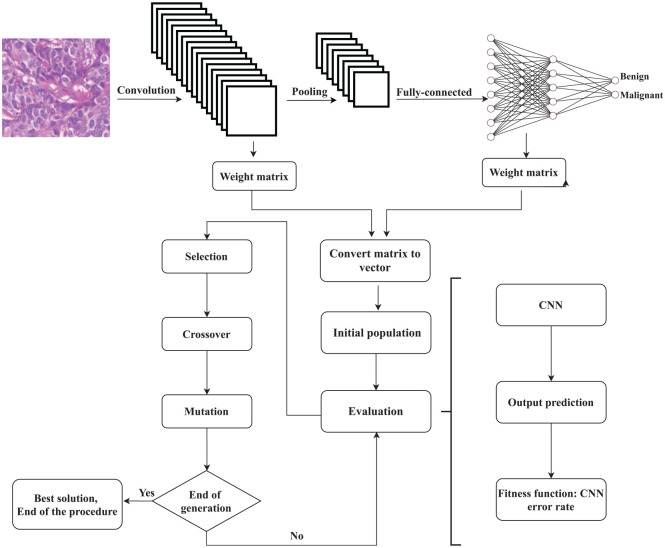
Block diagram of optimizing the parameters of the convolutional neural network (CNN) using the genetic algorithm.

In this section, we describe how we use the global search capability of the GA to evolve the CNN weights for the histopathological breast image classification problem. A CNN architecture suitable for binary classification of histopathological breast images is designed. Figure 2 shows a block diagram of our model. The layers used by the model are described below.

- *Input layer*: this layer loads the input images and passes them to the convolutional layer. In our design, inputs are breast biopsy images from the BreakHis dataset with dimensions of 210 × 210 × 3.
- *Convolutional layer*: this layer applies the convolution operation on input images using kernels (filters) to generate the feature map. The network consists of eight convolutional layers with multiple filters of size 3 × 3. The number of filters in each layer is as follows: eight filters for the first two layers, 16 filters for the third and fourth layers, 24 filters for the next two layers, and finally 32 filters for the last two layers. These filters of weights are initialized randomly using either uniform or normal distribution.
- *Pooling layer*: this step is performed after the convolution step. An average pooling function is applied to reduce the feature map dimension. This function calculates the average of each filter of the convolutional layer.
- *Activation layer*: all convolutional and pooling layers are then followed by a *ReLU* activation function (with the mathematical form of y=max(0,x) ) to add non-linearity to the model.
- *Fully connected layer*: after the convolution and pooling steps, the entire matrix of features is transformed into a single vector by applying a procedure called *flattening*. This vector is then fed to the fully connected layer of the network as its input neurons. In other words, the fully connected layer contains three layers: one input layer, one hidden layer, and one output layer. The input layer is a vector of features provided by the convolutional and pooling layers. The hidden layer resides between the input and output layers and transforms the input data to the output layer. Finally, the output layer is a vector of size two, which is the number of the classes in our binary classification, that is, benign and malignant classes.

After creating the CNN blocks, the whole system is trained. Training refers to the procedure of updating the weights of the network until the most accurate output is found. In our network, three different optimization techniques are utilized and compared in terms of classification accuracy: mini-batch gradient descent, the Adam optimizer, and the GA.

The GA evolves the population during run time and improves the solutions through several generations. The classification accuracy on the CNN highly depends on the weights in all layers. We improve this model using the GA. Since using matrix form makes the calculation of the CNN easier, all the weights of the network, including the weights of the convolutional filters and fully connected layers, are stored in a matrix for further computation.^17^ However, the initial population of the GA is stored in one-dimensional vectors. The weight matrix is converted to a vector to be used as the initial population of the GA, as indicated in the flowchart in Figure 2. This conversion is done using a Python function from the Numpy library, called *numpy.reshape*(•). The inputs of this function are the weight matrix and the size of the output vector. Thus, this vector will be used as the starting solution of the GA. A single solution of our model includes 26,125 weights. We have at least 20 solutions per population, which means a total minimum of 522,500 parameters.

Then to find the best set of the network weights, each solution is evaluated by a fitness function (the “Evaluation” block in Figure 2). That is, each set of weights is used to construct the corresponding neural network structure. The fitness of this solution is then calculated based on the classification error rate, as shown in Equation (3). After the evaluation step, the solutions are sorted based on their fitness values. Consequently, fitter individuals are selected to generate new offspring:

(3) $$\mathrm{Error}\;\mathrm{rate}=\frac{\mathrm{False}\;\mathrm{positives}+\mathrm{False}\;\mathrm{negatives}}{\mathrm{Total}\;\mathrm{number}\;\mathrm{of}\;\mathrm{samples}}$$

The selected solutions generated by the evaluation process are improved through the GA. The crossover and mutation operations of the algorithm are applied to selected solutions. A single-point crossover operator is performed on parent chromosomes to generate new offspring for the next generations. The mutation operation is used to change a single gene in each offspring aimed at increasing the diversity of the new generation. This operation adds a random value, generated using a uniform distribution, to a randomly selected gene. The iteration of evaluation, selection, crossover, and mutation is repeated (as shown in the flowchart) until the best set of the weights is found, producing the lowest classification error rate.

Algorithm 2 describes the algorithm for optimizing the CNN through the GA. We use the following parameters in the proposed GA optimizer implementation: (i) number of solutions per population varies between 20 to 60; (ii) number of generations is changed from 100 to 1000; (iii) mutation rate varies from 0.1 to 0.01; (iv) network weights are initialized randomly, using both normal and uniform distributions.

**Table:** 

**Algorithm 2** Optimizing the CNN through the GA.
Create the CNN architecture; Generate the initial weights of the model randomly; Store the weights of the model in a matrix; Set the parameters of the GA; Convert the weight matrix to the vector of initial population; **while** Termination condition is not true **do** Start the CNN process; Predict the output using the CNN; Calculate the fitness function using Equation (3); Sort individuals based on fitness value; Select individuals for the next generation; Crossover; Mutation; **end while**

## 5. Evaluation and dataset

We evaluated the performance of our proposed classifier on the BreakHis^20^ dataset. The performance analysis is conducted according to the evaluation metrics on the test set. We used different evaluation metrics, including classification accuracy, recall, precision, *F*_1_-score, and execution time. The main focus of this study will be on the classification accuracy metric. In what follows, we will describe the evaluation factors we used. Moreover, we will introduce the BreakHis dataset thoroughly.

### 5.1 Evaluation metrics

For evaluating the proposed model, we consider the basic performance measures derived from the confusion matrix.^49^ The confusion matrix is a table containing the outcomes of a binary classifier on the test data. The confusion matrix contains four components that are the outcomes of the binary classification:

- *TP*: true-positive prediction;
- *FP*: false-positive prediction;
- *TN*: true-negative prediction;
- *FN*: false-negative prediction.

Table 1 presents an overview of the confusion matrix for the breast cancer binary (benign and malignant) classification problem. The outcomes are different combinations of predicted and actual values.

**Table 1.** Confusion matrix for the breast cancer classification problem.

Actual Predicted	Benign	Malignant
Benign	*TN*	*FP*
Malignant	*FN*	*TP*

*TP*: true-positive prediction; *FP*: false-positive prediction; *TN*: true-negative prediction; *FN*: false-negative prediction.

#### 5.1.1 Accuracy

Classification accuracy is one of the important metrics to evaluate the performance of a classifier. This metric illustrates how accurate data will be classified using a particular classification model, as shown in Equation (4). The best classification accuracy is one, while the worst is zero^49^:

(4) $$\mathrm{Accuracy}=\frac{\mathrm{TP}+\mathrm{TN}}{\mathrm{TP}+\mathrm{TN}+\mathrm{FP}+\mathrm{FN}}$$

#### 5.1.2 Recall, precision, and *F*_1_-score

The breast cancer classification problem is an imbalanced classification problem. That is, the two classes we need to identify—benign and malignant—are different, and one is more important than the other, since we can probably tolerate false-positive predictions but not false-negative ones, because false-negative prediction means that the tumor is cancerous, while our classifier predicted it as a non-cancerous case. This wrong prediction may increase the treatment cost and cancer mortality rate. So, we need other metrics, such as recall and precision, which measure the relevance of the classification. Recall, also known as sensitivity in binary classification, illustrates the proportion of the total number of correct positive predictions and the total number of positive cases. A low recall rate means a large number of false-negative predictions. On the other hand, precision refers to the fraction of the total number of correct positive predictions and the total number of predicted positive cases. A low precision rate indicates a large number of incorrect positive predictions. Recall and precision are given by Equations (5) and (6), respectively^49,50^:

(5) $$\mathrm{Recall}=\frac{\mathrm{TP}}{\mathrm{TP}+\mathrm{FN}}$$

(6) $$\mathrm{Precision}=\frac{\mathrm{TP}}{\mathrm{TP}+\mathrm{FP}}$$

The balance between recall and precision can be calculated using a metric called the *F_1_-score*. The *F*_1_-score is a realistic measure to test the accuracy of the model by considering both recall and precision rates. The *F*_1_-score is given by the following equation:

(7) $$F_{1}-\mathrm{score}=2\times \frac{\mathrm{Recall}\times \mathrm{precision}}{\mathrm{Recall}+\mathrm{Precision}}$$

#### 5.1.3 Other metrics

Execution time is another performance metric to evaluate the proposed model. Execution time is defined as the total required time to complete a particular task by the central processing unit (CPU), without considering the input/output (I/O) waiting time or time required to complete other jobs. Besides considering the above-mentioned machine learning metrics, extensive experiments are performed for evaluating the impact of different GA parameters on the classification accuracy of the proposed classifier, such as the number of generations, number of solutions per population, mutation rate, and random number generator methods.

### 5.2 Dataset

**Figure 3.**
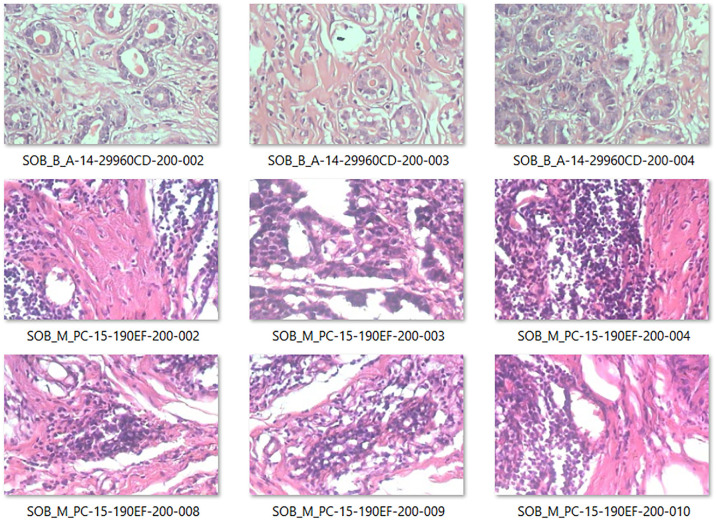
Slides of breast benign and malignant tumors with a magnification factor of 200X in the BreakHis dataset.

The BreakHis dataset, a public dataset available at http://web.inf.ufpr.br/vri/databases, is used to evaluate the performance of our proposed model for binary classification of histopathological breast images. Researchers need a unique dataset to evaluate the performance and prove the effectiveness of their proposed classifiers. Therefore, the BreakHis dataset, containing breast cancer histopathology images, was introduced by Spanhol et al.^20^ as a standard database for the breast cancer classification problem. Figure 3 indicates some samples of benign and malignant cases provided by the BreakHis dataset.

The BreakHis dataset includes 7909 breast cancer histopathological images, obtained from 82 patients. This dataset has both benign and malignant classes, which makes it a suitable choice for binary classification. Moreover, it contains multiple sub-classes of cancer types, such as Fibroadenoma, Adenosis, Phyllodes tumor, Tubular Adenona, Carcinoma, Lobular Carcinoma, and Papillary Carcinoma, which can be used for the multi-classification of breast biopsies. Also, the images are categorized based on their biopsy procedures as well as magnification factors (i.e. 40X, 100X, 200X, and 400X). The BreakHis dataset is widely used to design a valuable CAD system for the automated classification of breast biopsies.^3,11,31^

In this study, we randomly divided the BreakHis dataset into two sets, namely the training set and testing set, so that 70% of the existing images are used to train our classifier, and the remaining 30% are used to test the proposed model. These two sets are separated patient-wise, which means that patients used to create the training set and the test set are not the same. Moreover, as our focus is on the binary classification of breast histopathological images into benign and malignant cases, we are categorizing the breast biopsy images independent of their sub-classes and magnification factors.

## 6. Results

In this section, we present the results of histopathological breast image classification provided by the BreakHis dataset.

### 6.1. Accuracy

We train our proposed CNN model using the BreakHis dataset images as input and three different optimization approaches: mini-batch gradient descent, the Adam optimizer, and the GA. The output is the binary classification of the input images. Each image is passed to the network and labelled as a benign or malignant case. The predicted labels are then compared to the actual labels provided by the dataset to determine the classification accuracy. Then, the three discussed optimization techniques are evaluated by comparing the provided classification *accuracy*, *recall*, *precision*, *F_1_-score*, and *execution time* on an identical test set from the BreakHis dataset.

Classification accuracy is the metric to evaluate the performance of a classifier. The results shown in Table 2 present the highest classification accuracy achieved by each optimization approach. As the table demonstrates, the Adam optimizer, with 85.83% of the classification accuracy, outperforms the mini-batch gradient descent and GA methods, and mini-batch gradient descent provides lower classification accuracy than the other two optimizers. On the other hand, our proposed GA-based classifier performs almost as powerfully as the Adam optimizer, with a negligible difference. The best accuracy for mini-batch gradient descent and Adam is obtained when the batch size equal to 32, whereas the GA provides the best results with a batch of size 128.

**Table 2.** Classification accuracy of mini-batch gradient descent, Adam, and the genetic algorithm (GA) on the BreakHis dataset.

Optimizer	Batch size	Iteration	Accuracy
Gradient descent	32	300	69.88%
Adam	32	400	85.83%
GA	128	300	85.49%

To carry out this experiment, firstly, we ran the network using the mini-batch gradient descent technique to update the weights of the model. These weights are initialized randomly following a uniform distribution. The learning rate is set to 0.001. In the literature, the common learning rate used to train the CNN models varies from 0.1 to 0.0001. As 0.001 is introduced as a reasonable base learning value by some researchers,^51,52^ in this study we set the learning rate equal to 0.001. The weights of the model are updated after passing a batch of the training images through the network instead of a single image at a time. While a batch of size 32 is a good default batch value,^42^ we also tried higher batch sizes, that is, 64 and 128. Note that we experimented with these batch sizes following the work by Radiuk^53^ to study the impact of batch sizes on the proposed CNN model. The number of iterations is set between 100 and 1000 for this optimizer. The approach used to select this range was by starting from a small number of iterations and then increasing it gradually.

As can be seen from Table 2, the best classification accuracy obtained by mini-batch gradient descent is 69.88%. Using the same configuration, the Adam optimizer is then used to train the model. Finally, we trained our CNN model using the GA. The weights are initialized randomly by the uniform distribution. Solutions per population and number of parents mating are set to 40 and eight, respectively. To select these values, we followed what is used by Gad and Gad^17^ to optimize an ANN network using the GA. The difference is that since our CNN model has more parameters to update, we used a larger initial population. The number of parents mating is defined based on our training results. Since our experimental results indicated that in each iteration, almost 20% of solutions produce more acceptable classification accuracy, we decided to use them for producing offspring of the next generation.

Single-point crossover is used for offspring reproduction followed by a mutation operation, for adding a random value to a randomly selected gene, with a probability of 0.1. This value is recommended as a typical base value for mutation probability in the literature.^54^ The classification error rate is considered as the fitness function for the solution evaluation. These experiments are done in batches of size 32–128, for several generations between 100 and 1000. Since the results of the Adam optimizer and the GA are better than the gradient approach, their results are presented in detail in Table 3.

**Table 3.** Classification accuracy achieved by the Adam and genetic algorithm optimization approaches on the BreakHis dataset.

Batch size	Iteration	Adam accuracy	GA accuracy
32	100	68.73%	71.60%
32	200	63.20%	74.14%
32	300	84.53%	70.44%
32	400	85.83%	78.93%
32	500	83.01%	82.74%
32	1000	82.30%	82.14%
64	100	79.85%	74.90%
64	200	85.10%	75.17%
64	300	84.10%	80.30%
64	400	80.92%	85.01%
64	500	84.80%	84.15%
64	1000	82.74%	83.58%
128	100	83.56%	81.50%
128	200	85.63%	85.28%
128	300	84.46%	85.49%
128	400	85.69%	81.98%
128	500	84.11%	84.87%
128	1000	82.94%	82.97%

Table 3 illustrates that the Adam optimizer provides the best accuracy (85.83%) for a batch size of 32 and 400 iterations. For the GA, this is not the case. The batch size of 32 does not produce good accuracy unless the number of iterations increased to 1000. The best accuracy (85.49%) is obtained for 128 batches with 300 iterations. As can be seen, the GA performs equally well in comparison to the Adam optimizer for batch size 128. However, for other smaller batch sizes, the accuracy of the GA is better only for a larger number of iterations. For example, for 32 batches, compared to 70.44% for 300 iterations, 1000 iterations is 82.14%.

We can observe that for both optimizers, the overall classification improves for larger batch sizes. For a particular batch size, for smaller iterations, the accuracy is low. For example, for 100 iterations, batch size of 32 or 64, the accuracy is too low compared to a higher number of iterations for the same batch sizes. However, for the same number of iterations, 100, for batch size 128, the accuracy for both optimizers is close to those in higher iterations. From these observations, we can conclude that larger batch sizes provide better accuracy than smaller batch sizes.

The pattern we see in the table with larger batch sizes matches the results obtained by Radiuk,^53^ in which the impact of batch size on the performance of the CNN is studied. The author concludes that by increasing the batch size, accuracy will increase as well. We hypothesize that the reason for this improvement in accuracy and with increasing batch sizes could be due to the way in which the gradient of the loss function is calculated. When the batch size is a large number, a more accurate gradient can be obtained since we will update the weights after passing a large number of images through the model. Moreover, increasing the batch size decreases the chance of trapping in a local minimum, because the updates are done after training more images from the dataset. Consequently, these updates are globally good. Furthermore, the results demonstrate that, in general, increasing the number of iterations improves classification accuracy as well because more images are used to train the model, and the learning process is applied to the entire dataset many more times. As a result, the system learns better.

However, if we train the network with either a very high number of iterations or a too large number of batch sizes, the model will probably become over-fitted. Over-fitting refers to the situation in which the classifier does not generalize well between training data and test data. It means that there is a significant gap between training accuracy and test accuracy because the model did not learn the data and memorized it. As Table 3 shows, in most cases, by increasing the number of iterations, for example, from 500 to 1000 for batch size 128, the accuracy drops. We hypothesize that by increasing the number of batch sizes with increasing number of iterations, over-fitting would lead to poor accuracy.

**Figure 4.**
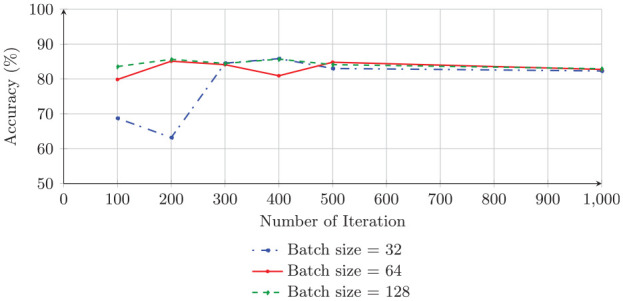
The testing accuracy of the trained convolutional neural network with Adam and batch size values of 32, 64, and 128 on the BreakHis dataset.

**Figure 5.**
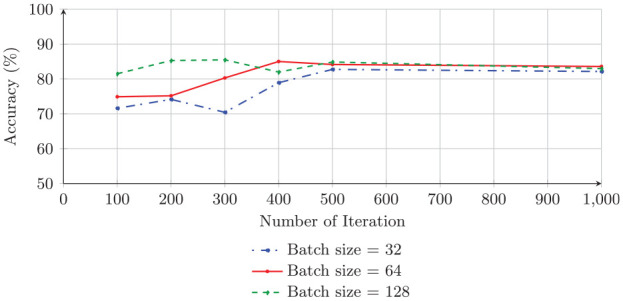
The testing accuracy of the trained convolutional neural network with the genetic algorithm and batch size values of 32, 64, and 128 on the BreakHis dataset.

Our proposed algorithm performs as well as the Adam optimizer and follows a similar pattern as that of the Adam optimizer. Unlike the Adam optimizer, the GA requires higher batch sizes for training the model. This is because, in general, in the GA, higher diversity and higher population size are needed to produce accurate results. It also requires a larger number of iterations to maintain stability. Therefore, this is not a surprise for this application. Thus, the GA requires a higher batch size (128) for training the model to gain the highest accuracy, 85.49%. Figures 4 and 5 show graphical representations of the results in Table 3 for both the Adam optimizer and the GA, respectively.

### 6.2 Recall, precision, and *F*_1_-score

As discussed above, the importance of the existing classes in the BreakHis dataset is not equal. Thus, in addition to measuring the classification accuracy, we need to evaluate our classifiers using *recall, precision*, and *F_1_-score* metrics, as well. To do so, the cases providing the highest classification accuracy for the Adam and GA optimization techniques are considered.

As Table 4 presents, the model using Adam and the GA on the BreakHis dataset obtained the highest accuracy compared to gradient descent, as we already discussed in the last section. Both optimizers also produce close values for recall, precision, and *F*_1_-score. Both Adam and the GA achieved a high precision rate: the precision for Adam and the GA are 96.23% and 94.71%, respectively. This implies that the number of false-positive predictions made by our model is significantly low, whereas the recall rate produced by either Adam or the GA is not as good as precision. This difference indicates that among wrong predictions, most of them are false-negative ones, which is not our preference. In other words, the model classifies the images with good accuracy and *F*_1_-score. However, among wrong predictions, there are more false-negative than false-positive cases, while we expected a better balance between them. To overcome this shortcoming, there are different ways; one possible solution could be decreasing the probability threshold in the loss function aimed at predicting positive cancer cases more often. This will be considered in future work.

**Table 4.** Comparison of the Adam and genetic algorithm techniques in terms of classification accuracy, recall, precision, and *F*_1_-score on the BreakHis dataset.

Optimizer	Accuracy	Recall	Precision	*F*_1_-score
Gradient descent	69.88%	55.61%	84.37%	67.02%
Adam	85.83%	72.31%	96.23%	82.56%
GA	85.49%	69.43%	94.71%	80.11%

Moreover, as shown in Table 4, the *F*_1_-score rates of all three classifiers are slightly less than their classification accuracy. However, for Adam and the GA, this rate is within an acceptable range. Overall, the results reveal that both Adam and the GA achieved acceptable accuracy and *F*_1_-score rates, which makes them capable for classification tasks.

### 6.3 Execution time

**Figure 6.**
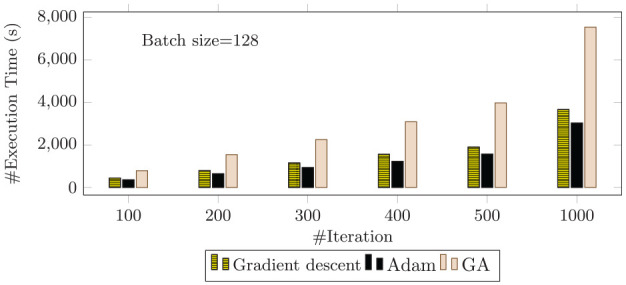
Bar chart comparing the execution time of gradient descent, Adam, and the genetic algorithm for different numbers of iterations and a constant batch size of 128.

In a clinical scenario, it is important to produce real time fast results. In this section, we therefore discuss the execution time of the proposed classifier. We implement the models in a sequential setting on a single processor. We experiment and discuss the total required time to complete a single classification task on a single processor. Figure 6 indicates the execution time for the three different classifiers: the gradient descent, Adam, and GA learning approaches, with a batch size of 128.

As presented in Figure 6, in all three classification models, increasing the number of iterations increases the execution time. More iterations implies more computation time to train the system using the images. Therefore, it is reasonable to expect an increase in execution time for a greater number of iterations. Thus, for all models, there is an increase in execution time for 500 to 1000 iterations, which is consistent with the fact that more iterations imply more computations. For gradient descent, although the execution time is lower compared to the GA, it does not produce good accuracy. So, the gradient descent approach can be ignored. The comparison is basically between Adam and the GA.

The GA explores the solution space with an initial population and randomly generates other fitter solutions over a period of time. In the model, we first randomly generate the initial weights. Since we consider multiple solutions per population, the total number of parameters increases significantly. As a result, more time is required to complete the breast cancer classification task using the GA. This is consistent with the literature on the GA.^55^ To expedite the GA process, it is important to execute the algorithm on parallel machines.^56,57^ Since this study was to demonstrate the feasibility of using the GA for training the CNN for the histopathological breast image classification problem, parallelization was not considered in this study.

### 6.4 GA parameters

We also conducted other experiments to understand the GA a little better; in particular, the impact of the different GA parameters on the accuracy of the proposed classifier. We considered parameters such as the initial population, random number generator methods, and mutation rate. Classification accuracy is used for performance comparison.

Finding the proper size of the initial population is the primary step in running a GA. Small or large population sizes may lead to a poor final solution, while using the proper size increases the chance of finding a more accurate solution.^58–60^ The proper number of starting solutions is a number that guarantees enough diversity in the whole population. Therefore, we considered the impact of varying the initial population size on the classification accuracy of histopathological breast images. In this experiment, we considered the results for 300 and 400 iterations.

Table 5 indicates that the final results achieved by the initial population of size 20 are not as good as either starting population size of 40 or 60. The reason for this weakness could be due to the lower diversity and search space of the initial solutions. In this work, we considered an initial population of size 40 to start searching for the best CNN weights, since it provides good accuracy and needs less computational resources and execution time, rather than a population size of 60 or higher.

**Table 5.** The effect of initial population size on classification accuracy of the genetic algorithm.

Classification accuracy				
Batch	Iteration	Population size = 20	Population size = 40	Population size = 60
32	300	69.56%	70.44%	82.19%
32	400	76.32%	78.93%	80.97%
64	300	81.74%	80.30%	78.61%
64	400	70.21%	85.01%	84.80%
128	300	76.49%	85.49%	72.21%
128	400	80.73%	81.98%	85.24%

**Figure 7.**
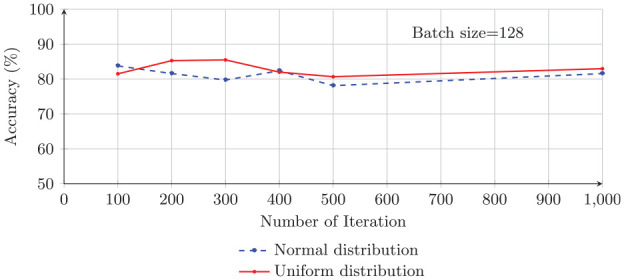
Testing the classification accuracy of the genetic algorithm optimizer using normal and uniform distributions on batch size values of 32, 64, and 128.

The GA takes a random initial population of possible solutions and evolves them slowly until it finds the best solution. Thus, the random number generator methods may influence the quality of the final results.^61^ We investigate the impact of normal and uniform distributions to find the best set of initial network weights by the GA. Figure 7 represents how classification accuracy changes by using normal and uniform distributions.

In general, the classification accuracy achieved by uniform distribution, to generate the initial weights of the CNN model, is slightly better than the normal distribution. This result coincides with what is mentioned by Maaranen et al.^62^ about the random generation of the initial population in the GA. As discussed in this study, the uniform distribution contains diverse points, which makes it a useful technique to generate the initial population of the GA when there is no prior knowledge about the final result.

Finally, we investigated the impact of the mutation operation on the classification accuracy of the proposed GA-based CNN model. Like other GA parameters, mutation probability^63^ has an influence on the quality of the final results. Lynch et al.,^63^ in their article, discuss the influence of mutation on population diversity, on improving replication, and also the adverse effect of mutations in the population. This mutation rate determines the number of solutions that need to be mutated in each generation to generate new offspring. Mutation is applied in applications to help the GA avoid trapping in a local minimum. If we ignore the mutation operation after applying crossover, the chances of getting stuck in the local minimum increase, since diversity is not considered. The mutation function solves this problem by producing offspring different from good parents and encouraging the diversity of the solutions.^47^

While a very high mutation probability may prevent the convergence of the population to a good solution, very small probability can lead to premature convergence, since just good offspring are produced by the good part of the parents after applying crossover. Our model is tested using mutation rates of 0.1 and 0.01, as shown in Table 6. As we have the highest accuracy when the mutation rate is 0.1, we considered this mutation probability for all other experiments.

**Table 6.** Testing the impact of mutation probability on the classification accuracy of the genetic algorithm.

Classification Accuracy			
Batch	Iteration	Mutationrate = 0.01	Mutationrate = 0.1
32	100	69.84%	71.60%
32	200	63.20%	74.14%
32	300	76.95%	70.44%
32	400	74.22%	78.93%
32	500	81.38%	82.74%
32	1000	80.91%	82.14%
64	100	71.88%	74.90%
64	200	76.54%	75.17%
64	300	83.52%	80.30%
64	400	82.06%	85.01%
64	500	78.93%	84.15%
64	1000	82.36%	83.58%
128	100	84.74%	81.50%
128	200	81.29%	85.28%
128	300	82.46%	85.49%
128	400	80.95%	81.98%
128	500	83.07%	84.87%
128	1000	78.23%	82.97%

## 7. Discussion and conclusion

Breast cancer is the most regularly diagnosed cancer and the main cause of cancer mortality in women around the world. CAD systems have become essential and crucial in the breast cancer classification problem. Machine learning techniques, such as CNNs, because of their classification capabilities, have been widely adopted in CAD systems. The precision of extracted features in the CNN is highly dependent on the weights of the network, including the weights of all convolution filters, as well as the weights of connecting edges in the fully connected layer. As a result, these weights play a crucial role in classification accuracy. In this study, we proposed to optimize the weights of the CNN using the GA for the histopathological breast image classification problem. The GA, a well-known global optimization technique, has been widely used for many real-world applications. However, there is very little work on combining nature-inspired computing with machine learning. This study attempted to do this. We provide some insight into the feasibility of hybridizing the CNN with the GA for the histopathological breast image classification problem.

The proposed algorithm consisted of five steps (Figure 2): the input layer, convolution layer, pooling layer, activation layer, and fully connected layer, and the training process. The classification accuracy highly depends on the weights of these layers. The weights are evolved using selection, crossover, and mutation operators. We compared our proposed method with the mini-batch gradient descent approach and the Adam optimizer using the BreakHis dataset. We performed various experiments on different batch sizes and the number of iterations to study metrics such as accuracy, recall, precision, *F*_1_-score, and execution time, as well as the effect of genetic parameters on the proposed algorithm.

Our experimental results indicated that among the three classifiers, the mini-batch descent classifier provided lower accuracy. We showed that the proposed GA-based classifier is as good as the Adam optimizer but with larger batch size and iterations. In general, we observed that the classification accuracy improves for Adam and our proposed classifier for larger batch sizes. This observation coincides with the observations of Radiuk.^53^ Larger batch sizes improve the training process by using more images to update the weights and also prevent the model from getting trapped in local optima.

We also noted that both Adam and the GA achieved a higher precision rate. This implies that the number of false-positive predictions made by our model is significantly low (less than 0.1). However, the recall metric result is not satisfactory for either optimizer. The *F*_1_-score is an acceptable rate.

The total execution time for the GA is higher than that for the other two optimizers. Since the GA is an evolutionary process, the stability and accuracy are dictated by the selection, crossover, and mutation process over a period of time. In line with the literature,^63^ the population size affected the accuracy of the GA. A larger population size produced better results. The classification accuracy achieved by uniform distribution to generate the initial weights was slightly better than that achieved by the normal distribution.

In conclusion, the proposed GA classifier is a viable method in evolving the weights of the network. Combining the strengths of machine learning and evolutionary algorithms (evolutionary machine learning) is a promising research to pursue for real-world applications.

## 8. Future work

This research studied the feasibility of hybridizing an evolutionary algorithm with machine learning models. Evolving the CNN using the GA is a promising approach for an important problem. As part of the future work, we propose here some ideas to enhance our current model.

- Incorporating diversity: currently, we have one single population on a single “island.” The crossover and mutation are performed on this island. Diversity is important in the GA for better accuracy. We propose to use the Island GA model, which is a multi-population technique in which chromosomes migrate between existing sub-populations (or islands) to increase the diversity. This technique may prevent early convergence by increasing the population diversity to achieve higher classification accuracy. We propose to study various island topologies to provide different cross-fertilization. We believe this approach can accommodate smaller batch sizes, as in the Adam optimizer, since the population per island and number of islands can be controlled to suit the batch sizes.
- Improving computation time: we propose to parallelize the island model. Each island could be implemented on a different processor, providing a great deal of concurrency and thereby improving the performance.
- Improving initial population—collaborative approach: in this approach, we propose to initially use the Adam optimizer to train the model. We will then pass the best set of network weights found by the Adam optimizer to the GA to initialize its population. The GA will then evolve the solutions, which may provide better accuracy and faster convergence.
- Improving the performance metric recall: one possible solution to improve the recall value is to decrease the probability threshold in the loss function. This will help in predicting positive cancer cases efficiently, reducing the rate of false-negative results and thereby decreasing the treatment cost and cancer mortality rate.
